# A method for detecting small objects in remote sensing images based on feature enhancement and scale-adaptive optimization in complex scenarios

**DOI:** 10.1371/journal.pone.0350873

**Published:** 2026-10-27

**Authors:** Xiaoxiao Wang, Xia Zou, Meng Sun, Chong Jia, Yongqiang Xie, Xiongwei Zhang

**Affiliations:** 1 Army Engineering University of PLA, Nanjing, China; 2 State Key Laboratory of Advanced Communication Technology, Nanjing, China; 3 Chinese People’s Liberation Army Unit, Beijing, China; Nanjing Forestry University, CHINA

## Abstract

To address the challenges of small object detection in remote sensing images, such as susceptibility to background noise and difficulties in feature extraction, this paper proposes a method for detecting small objects in remote sensing images based on feature enhancement and scale-adaptive optimization under complex scenarios. First, we constructed a bidirectional complementary feature fusion network, employing a bidirectional feature fusion mechanism to enhance the interaction between shallow-level details and deep-level semantic features, thereby enabling more effective multi-scale feature fusion. Second, we developed a Multi-scale Local-Global Attention (MLGA) module that uses multi-scale pooling to capture both local details and global context, and employs dynamic weights for adaptive feature fusion, effectively suppressing background noise and highlighting the features of small objects. In addition, we designed a scale-adaptive bounding box regression loss function (SAWIoU) that addresses the scale bias issue for small objects by introducing a scale scaling factor, thereby ensuring balanced optimization across objects of different scales and further improving localization accuracy. Finally, experimental results on two public remote sensing datasets DIOR and RSOD show that the proposed method achieved mAP50 scores of 0.885 and 0.971, respectively, significantly outperforming current mainstream detection models, with particularly notable improvements in small-object detection accuracy. Additionally, ablation studies further validate the effectiveness and robustness of this method in detecting small objects in remote sensing under complex backgrounds.

## 1 Introduction

Remote sensing technology, with its advantages of wide coverage and non-contact operation, has been widely applied in fields such as disaster monitoring, environmental observation, and urban planning, providing crucial data support for target identification and dynamic perception [1,2]. Consequently, remote sensing image detection has emerged as a key technology for achieving automatic target extraction and efficient information analysis, and its importance is becoming increasingly prominent [3].

In recent years, deep learning has greatly advanced object detection in remote sensing images. Among general-purpose one-stage detectors, the YOLO series has evolved from YOLOv5, which established a foundation for industrial applications, to YOLOv8 with an anchor-free mechanism, and further to the latest versions, YOLOv11 and YOLOv12, which incorporate regional attention and efficient gradient flow optimization. These developments have continuously improved feature representation capability and inference efficiency. Meanwhile, researchers have also explored specialized detection schemes tailored to the inherent properties of remote sensing images. For example, Liu et al. [4] developed an Adaptive Balanced Network (ABNet) for object detection in remote sensing images; Zhang et al. [5] proposed SFSANet, a multi-scale object detection network for remote sensing images based on semantic fusion and scale adaptability. In terms of context information mining, Zhu et al. [6] fused global and local context information and introduced an adaptive anchor module to design GLNet, which balances macro-level environments and micro-level details. Liu et al. [5] further proposed SRAF-Net, an anchor-free detection network for remote sensing images. Furthermore, Zhang et al. [7] developed a new object detection network, CoF-Net, using a progressive strategy to achieve feature extraction. However, remote sensing images are typically acquired from high-altitude platforms. Due to limitations in imaging distance and spatial resolution, objects often occupy only a limited number of pixels in an image. In particular, small objects (such as vehicles, ships, and aircraft) are usually composed of only a few dozen or even just a few pixels, resulting in weak visual characteristics and increased susceptibility to noise interference. Consequently, directly applying general-purpose detectors often fails to achieve satisfactory results [8,9].

To address the challenge of detecting small targets in remote sensing images, where targets are extremely small and susceptible to interference from complex backgrounds, existing research has conducted systematic investigations across multiple dimensions, including feature fusion, feature extraction, and optimization strategies [10,11]. In addition, recent studies on deep learning and feature learning have shown that adaptive feature representation, cross-level interaction, and degradation-aware modeling are effective for improving visual perception in complex scenes [12–15]. In particular, multi-scale feature fusion effectively mitigates the issue of small objects being easily lost in deep-level features by integrating semantic information and spatial details across different levels. Attention mechanisms enhance the model’s ability to distinguish features by amplifying responses in target-relevant regions and suppressing redundant background information. Improvements to the loss function primarily focus on optimizing imbalance and error metrics during classification and localization, thereby further improving bounding box regression accuracy and detection robustness. The aforementioned research achieves targeted improvements in small-object detection performance from various perspectives, and their synergistic effects constitute current major research directions in this field.

Multi-scale feature fusion enhances a model’s ability to represent small objects by deeply exploring cross-level semantic relationships and fine-grained spatial information. Methods such as the Feature Pyramid Network (FPN) construct top-down feature propagation paths to effectively inject high-level semantic information into shallow, high-resolution features, thereby significantly improving the detectability of small objects [16]. On this basis, the Path Aggregation Network (PANet) introduces bottom-up enhancement paths to shorten the distance for propagating low-level detail information to higher levels, thereby achieving more comprehensive bidirectional feature fusion [17, 18]. On the other hand, the Bi-directional Feature Pyramid Network (BiFPN) employs weighted bidirectional fusion and cross-scale connections to enhance the interaction of multi-scale information while maintaining computational efficiency [19, 20]. The Neural Architecture Search-based FPN (NAS-FPN) optimizes the fusion structure through automatic neural architecture search, thereby further enhancing the flexibility and expressiveness of fusion strategies [21,22]. In addition, the Adaptive Structure Feature Fusion (ASFF) introduces learnable weights to adaptively fuse features of different scales in the spatial domain, enabling the model to dynamically select the optimal feature representation based on scene content [23,24]. In summary, the above methods enhance the effective integration of multi-scale information from different perspectives. To some extent, they alleviate the detection difficulties caused by scale variations of small objects and interference from complex backgrounds.

Attention mechanisms simulate the selective focus characteristic of the human visual system. These mechanisms guide the network to focus on target-related regions and suppress redundant background information, thereby improving the model’s feature discrimination capability. The channel attention mechanism, represented by Squeeze-and-Excitation Networks (SENet), uses global average pooling to model dependencies among channels and adaptively recalibrates feature responses, thereby significantly enhancing feature extraction capabilities [25]. Building on this, the Convolutional Block Attention Module (CBAM) further integrates spatial attention, enabling joint modeling of both channel and spatial dimensions. This allows the network to simultaneously focus on “which features are important” and “where the target is located” [26,27]. To address the challenges of small target scales and the loss of positional information in remote sensing small object detection, the Coordinate Attention (CA) mechanism embeds positional information into channel attention. It enhances spatial location awareness while keeping the model lightweight [28,29]. Meanwhile, the SimAM employs a parameter-free 3D attention model that uses an energy function to evaluate neuron importance, further reducing computational overhead [30,31]. The aforementioned methods enhance the model’s ability to distinguish target features from various dimensions. They play a key role in suppressing complex background interference, thereby providing important support for remote sensing small-object detection.

The design of the loss function directly determines the optimization direction and convergence properties of the network during object localization. The traditional Intersection over Union (IoU) loss focuses solely on the overlapping region between the predicted bounding box and the ground-truth bounding box. When no overlap exists between the two boxes, the loss fails to provide effective gradient information, thereby limiting the model’s optimization capabilities. To address this, the GIoU loss introduces a penalty term for the minimum bounding box, enabling it to provide effective gradient propagation even in cases of non-overlap [32]. Based on this foundation, the DIoU loss further accounts for the distance between the center points of the predicted and ground-truth bounding boxes, accelerating the convergence process of bounding box regression [33]. The CIoU loss additionally incorporates an aspect ratio consistency constraint to enhance the precision of localization results [33]. To address the issues of large-scale sensitivity and easily amplified localization errors in small-object detection, recent research has further introduced dynamic sample adjustment mechanisms. Among these, the WIoUv3 loss characterizes sample quality through outlierness and constructs a non-monotonic focus coefficient. This allows the loss function to adaptively assign gradient weights to samples of different qualities, thereby effectively improving localization accuracy and detection robustness for small objects [34]. Therefore, the continuous evolution of loss functions provides important support for the precise localization of small objects.

Although the aforementioned methods have achieved significant progress at various levels, several key bottlenecks remain in the task of small-object detection in remote sensing images with complex backgrounds. First, at the feature fusion level, while multiscale fusion has been widely adopted, mainstream feature pyramid architectures largely rely on unidirectional or simple bidirectional information flow. The interaction between shallow details and deep semantics remains insufficient, thereby limiting effective complementarity among multi-scale features. This limits the feature representation capability for small objects. Second, at the feature extraction level, existing attention mechanisms struggle to balance local details and global context information. Global modeling helps capture overall semantic information but may weaken the already subtle responses of small objects. In contrast, local modeling is sensitive to background noise, which can easily cause an attention shift. Finally, at the localization optimization level, IoU-based loss functions exhibit inherent scale sensitivity toward small targets. Due to the small area of the target, even if the predicted box has high localization accuracy, its IoU value may still be low. Consequently, such samples are misclassified as low-quality in sample quality assessments (such as the outlier metric in WIoUv3), leading to suppressed gradient contributions and ultimately affecting localization accuracy. Overall, these issues often occur simultaneously, collectively limiting further improvements in the performance of small-target detection in complex backgrounds.

To address the aforementioned issues, this paper proposes a Feature-enhanced and scale-adaptive optimization network (FESANet) for small object detection in remote sensing images with complex backgrounds. First, a Reciprocal Complementary Feature Fusion (RCFF) network is constructed using a bidirectional feature interaction mechanism. Through gated fusion and adaptive recalibration between low-level and high-level features, RCFF enhances the effective complementarity between shallow-level details and deep-level semantics, thereby addressing the shortcomings of traditional feature pyramids in information exchange. Second, a Multi-scale Local-Global Attention (MLGA) module is developed. This module uses multi-scale pooling to capture local details across different receptive fields and combines them with global contextual information for joint modeling. Employing dynamic weights to achieve adaptive feature fusion, it enhances the feature responses of small objects while suppressing interference from complex backgrounds. Furthermore, we designed a Scale-Adaptive WIoU (SAWIoU) loss function. By introducing a scale scaling factor to correct the IoU, this loss mitigates scale bias issues for small objects, making sample quality evaluation more reasonable. This ensures that the dynamic focusing mechanism achieves balanced optimization across targets of different scales, thereby further improving localization accuracy. Finally, we validate the effectiveness and superiority of FESANet on two datasets: DIOR and RSOD. The main contributions of this study are as follows:

(1) We construct a reciprocal complementary feature fusion network to strengthen the interaction between high- and low-level features and enhance the expressive power of multi-scale features.
(2) A multi-scale local–global attention mechanism is designed to effectively suppress background interference and enhance the representation of small-object features by integrating local details with global contextual information.
(3) A scale-adaptive loss function is proposed to improve small-object localization accuracy by correcting IoU scale bias and optimizing sample weight allocation.

## 2 Methods

The overall framework of the proposed FESANet is illustrated in Fig 1. It was implemented based on the official Ultralytics YOLOv11 detection framework. Specifically, YOLOv11n was adopted as the baseline model, and the proposed RCFF, MLGA, and SAWIoU modules were integrated into its neck network, attention-enhanced feature representation, and bounding-box regression loss. The original backbone and detection head of YOLOv11n were kept unchanged to ensure a fair comparison. Specifically, the RCFF module enhances multi-scale feature representation via bidirectional feature interaction and gated high‑low level fusion. The MLGA module improves discriminability for small objects under complex backgrounds by combining multi‑scale adaptive pooling with global contextual modeling. The SAWIoU loss function refines localization accuracy through scale‑corrected IoU and an optimized dynamic focusing mechanism. Working in synergy, these three modules collectively deliver a comprehensive improvement in detection performance for small objects in complex remote sensing scenes.

**Fig 1:**
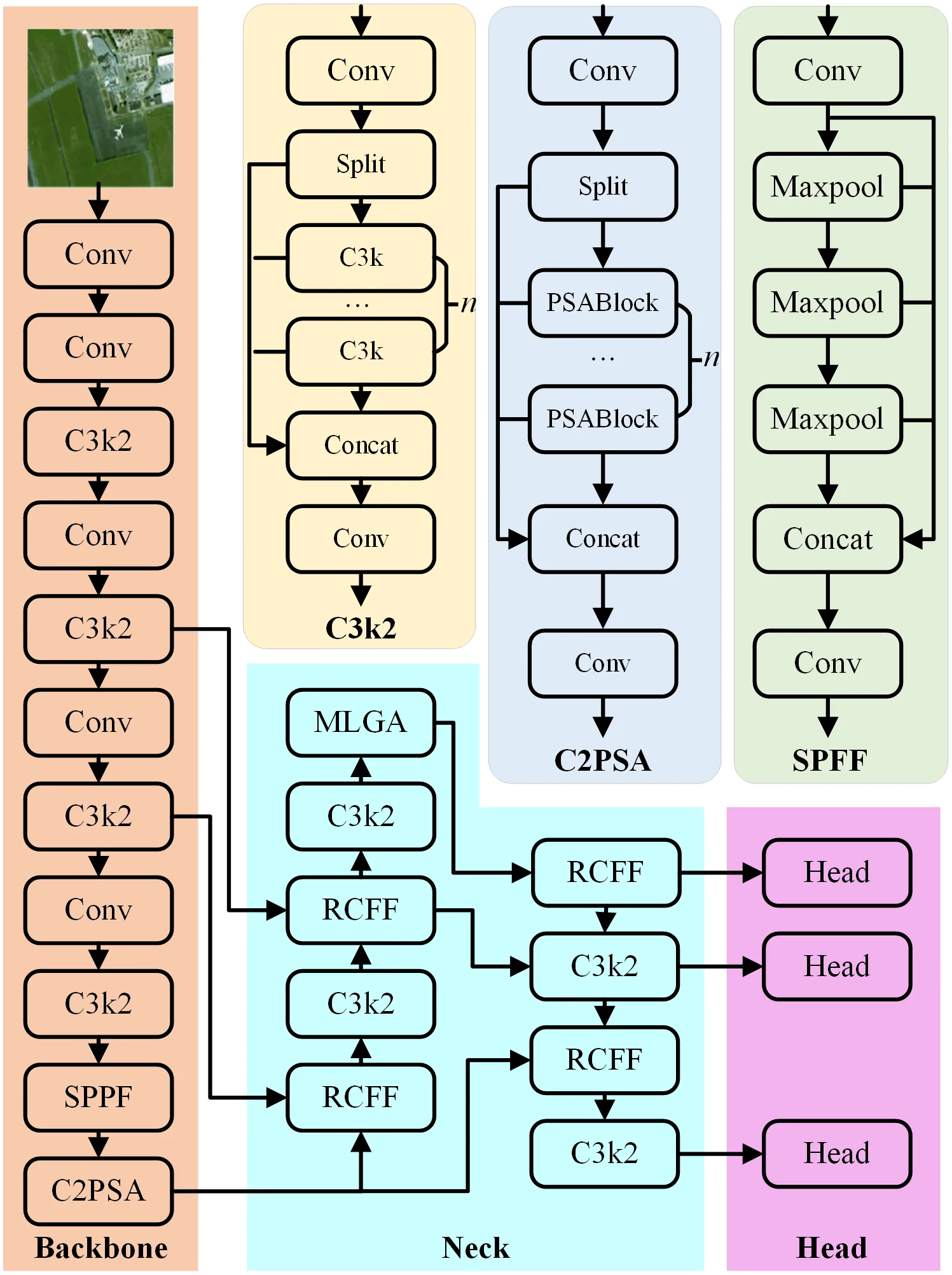
Overall architectural framework of FESANet.

### 2.1 Reciprocal complementary feature fusion network

For small objects in remote sensing images under complex backgrounds, high-level features undergo multiple downsampling operations, which easily leads to “semantic degradation” of their weak spatial information. In contrast, low-level features retain high-resolution details but lack sufficient global context to suppress background noise. To enhance effective cross-layer information interaction and improve the representation capability of multi-scale features for small objects, this paper constructs the RCFF network, whose structure is shown in Fig 2. This network introduces an adaptive gating mechanism that enhances single-layer features while selectively “borrowing” missing information from adjacent feature layers, thereby enabling effective cross-scale feature complementarity.

**Fig 2:**
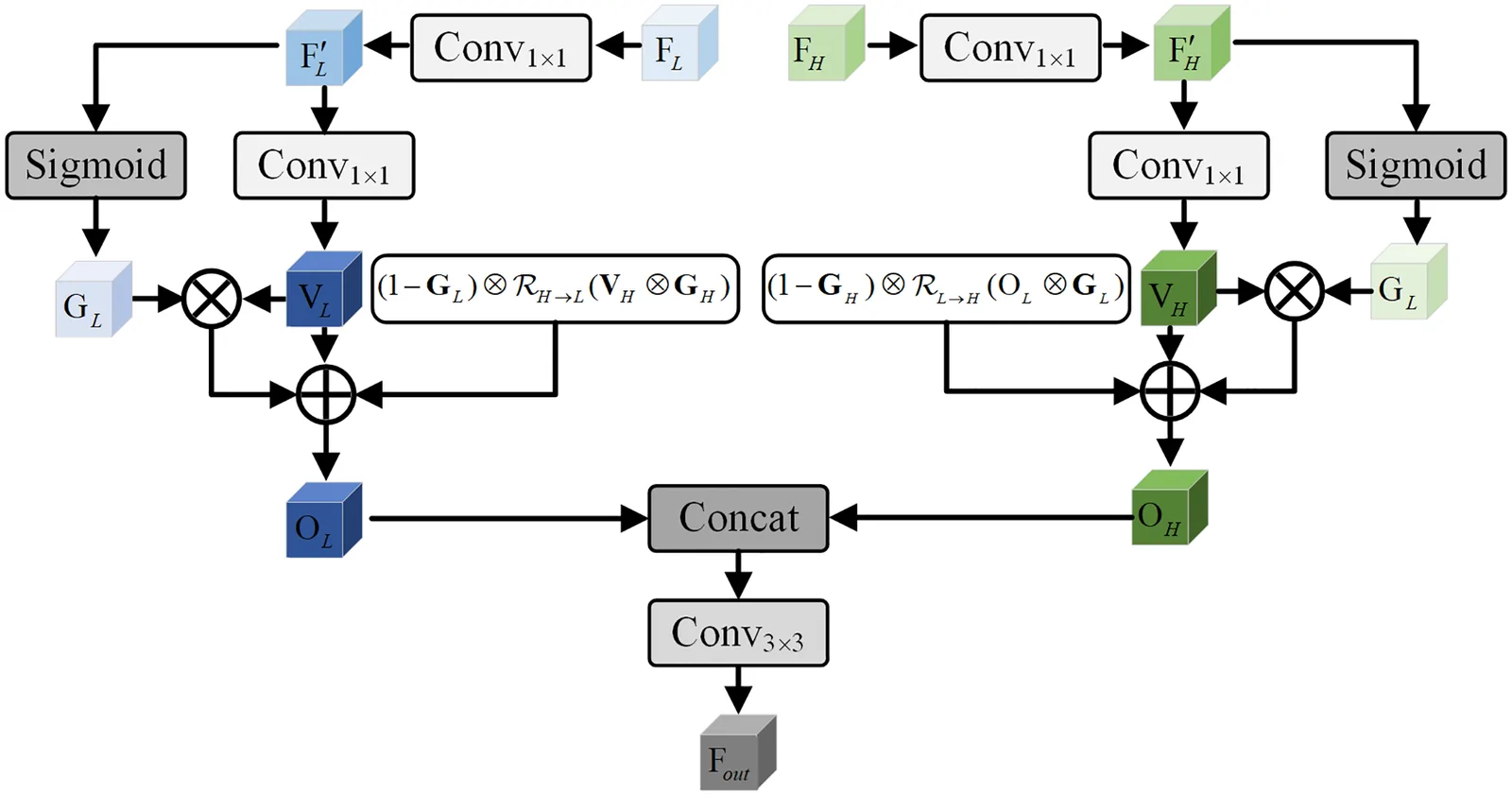
Reciprocal complementary feature fusion network architecture.

Let the adjacent high-level and low-level features from the feature pyramid be denoted as ${𝐅}_{H}$ and ${𝐅}_{L}$, respectively. To reduce computational complexity and align the channel dimensions, RCFF first compresses the number of channels of both feature maps to the target dimension using 1 × 1 convolutions:


$$\begin{array}{c} \begin{array}{c} {{𝐅}^{\prime }}_{H}={\text{Conv}}_{1\times 1}\left({𝐅}_{H}\right)\\ {{𝐅}^{\prime }}_{L}={\text{Conv}}_{1\times 1}\left({𝐅}_{L}\right) \end{array} \end{array}$$ (1)

Subsequently, the corresponding soft attention gating masks ${𝐆}_{H}$ and ${𝐆}_{L}$ are generated along the spatial dimension using the Sigmoid activation function to evaluate the saliency of information at different spatial locations within the current feature layer. Meanwhile, a nonlinear mapping is applied again to the dimension-reduced features ${{𝐅}^{\prime }}_{H}$ and ${{𝐅}^{\prime }}_{L}$ to obtain the feature representations ${𝐕}_{H}$ and ${𝐕}_{L}$ for subsequent fusion.

After obtaining the gating masks for each branch, the module adopts a bidirectional information propagation strategy that combines “self-enhancement and complementary borrowing.” Taking the update of low-level features as an example, its mathematical expression is as follows:


$$\begin{array}{c} {𝐎}_{L}={𝐕}_{L}+\underset{\text{Self-enhancing}}{\underbrace{{𝐕}_{L}⊗{𝐆}_{L}}}+\underset{\text{Selective borrowing}}{\underbrace{\left(1-{𝐆}_{L}otimes{ℛ}_{H\to L}({𝐕}_{H}⊗{𝐆}_{H}\right)}} \end{array}$$ (2)

where, ⊗ denotes element-wise multiplication, and ${ℛ}_{H\to L}(\cdot )$ represents the operation of aligning the spatial resolution of high-level features through bilinear interpolation. The self-enhancement strategy uses the feature’s own gating mask to strengthen salient regions with strong responses in low-level features (e.g., local textures of the target). When the low-level feature has a weak response in a certain region (i.e., ${𝐆}_{L}$ approaches 0, making $1-{𝐆}_{L}$ large) and may suffer from missing information, the network adaptively supplements that region with semantic features ${𝐕}_{H}⊗{𝐆}_{H}$ filtered by the high-level gating mechanism.

Similarly, high-level features are updated symmetrically in a cascaded manner for cross-layer complementary enhancement:


$$\begin{array}{c} {𝐎}_{H}={𝐕}_{H}+\underset{\text{Self-enhancing}}{\underbrace{{𝐕}_{H}⊗{𝐆}_{H}}}+\underset{\text{Selective borrowing}}{\underbrace{\left(1-{𝐆}_{H}otimes{ℛ}_{L\to H}({𝐎}_{L}⊗{𝐆}_{L}\right)}} \end{array}$$ (3)

When a small object region in the high-level feature map is lost due to downsampling, the corresponding ${𝐆}_{H}$ becomes smaller. The network then assigns a larger weight to $1-{𝐆}_{H}$, thereby accurately extracting high-resolution boundary and localization information from the low-level features ${𝐎}_{L}$ to complete feature reconstruction.

Following the selective interaction described above, high-level and low-level features have achieved sufficient cross-scale information exchange at both the semantic and spatial scales. Finally, the updated high-level features ${𝐎}_{H}$ are upsampled to the same resolution as the low-level features ${𝐎}_{L}$, concatenated along the channel dimension, and then undergo channel fusion and feature smoothing through a 3 × 3 convolutional layer, resulting in the final enhanced feature map ${𝐅}_{out}$:


$$\begin{array}{c} {𝐅}_{out}={\text{Conv}}_{3\times 3}\left(\text{Concat}\left[{ℛ}_{H\to L}\left({𝐎}_{H}\right),{𝐎}_{L}\right]\right) \end{array}$$ (4)

### 2.2 Multi-scale local-global attention mechanism

The MLGA module is designed to capture both global contextual information and multi-scale local details simultaneously. By fusing these two components through adaptive weighting, the model can dynamically adjust the attention distribution based on the input image, thereby enhancing features of small objects and suppressing background noise. Its structure is shown in Fig 3.

**Fig 3:**
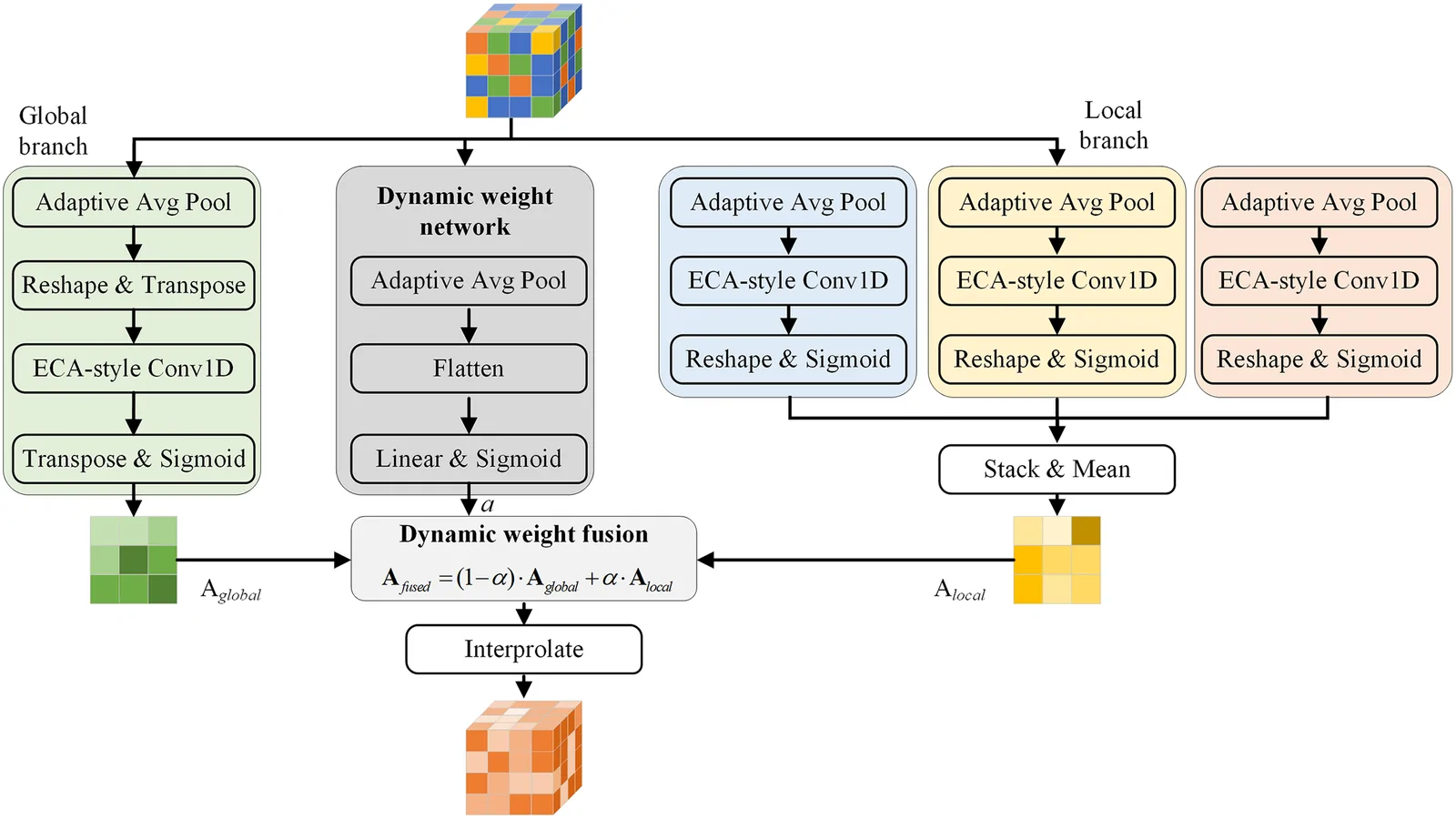
Multi-scale local-global attention mechanism.

Let the input feature map be $𝐗\in {\mathbb{R}}^{C\times H\times W}$, where *C*, *H*, and *W* represent the number of channels, height, and width, respectively. MLGA first divides the data into two parallel branches—global and local—for processing.

To capture dependencies across channels within the global receptive field, we first perform global average pooling on the spatial dimensions of **X**. We then adopt the approach used in the Efficient Channel Attention mechanism to facilitate cross-channel information exchange via one-dimensional convolutions. To mitigate information loss caused by dimensionality reduction, the kernel size k of the one-dimensional convolutions can be adaptively calculated based on the number of channels:


$$\begin{array}{c} k=\phi \left(C\right)={\left|\frac{{log}_{\text{2}}\left(C\right)}{\gamma }+\frac{b}{\gamma }\right|}_{odd} \end{array}$$ (5)

where, γ and b are hyperparameters (this paper defaults to γ=2,  b=1), $|\cdot {|}_{odd}$ denotes taking the nearest odd number. The calculation formula for the global attention weight ${𝐀}_{global}$ is:


$$\begin{array}{c} {𝐀}_{global}=\sigma \left({\text{Conv1D}}_{k}\left({𝒫}_{GAP}\left(𝐗\right)\right)\right) \end{array}$$ (6)

where, σ(·) is the Sigmoid activation function, and ${𝒫}_{GAP}$ represents the global average pooling operation.

At the same time, to capture local texture and boundary information of small objects across different receptive fields, the MLGA module introduces a set of multi-scale local pooling operations. Specifically, a set of local pooling scales S={3,5,7} is defined, and adaptive average pooling is applied to the input feature **X** to generate local feature representations ${𝐅}_{s}\in {\mathbb{R}}^{C\times s\times s}$ at the corresponding scales. Subsequently, while preserving spatial structure, a one-dimensional convolution with a shared kernel size *k* is applied along the channel dimension for features at each scale, thereby obtaining multi-scale local channel attention maps:


$$\begin{array}{c} \begin{array}{c} {𝐀}_{local}^{\left(s\right)}=\sigma \left({\text{Conv1D}}_{k}\left({𝐅}_{s}\right)\right),\;\;\;\;\forall s\in S\\ {𝐀}_{local}=\frac{\text{1}}{\left|S\right|}\sum_{s\in S}{𝐀}_{local}^{\left(s\right)} \end{array} \end{array}$$ (7)

After aligning the local attention maps from different scales along the spatial dimension, we perform mean fusion to obtain the final local attention representation ${𝐀}_{local}$. By preserving explicit local spatial dimensions, this branch effectively prevents small-scale features from being diluted by the vast amount of background information in remote sensing images.

Furthermore, in remote sensing scenarios, due to the constantly changing complexity of the background in which targets are located (such as distinct road surfaces and complex urban building clusters), the balance between global background suppression and local feature enhancement must be dynamically adjusted. To this end, MLGA has designed a feature-context-based dynamic weight generation network. By analyzing global features, this network outputs an adaptive balancing factor α:


$$\begin{array}{c} \alpha =\sigma \left({𝐖}_{d}\cdot \text{Flatten}\left({𝒫}_{GAP}\left(𝐗\right)\right)+{𝐛}_{d}\right) \end{array}$$ (8)

Here, ${𝐖}_{d}\in {\mathbb{R}}^{C}$ represents the trainable weights of the fully connected layer. Using this balance factor α, the network adaptively fuses global and local attention through weighted averaging:


$$\begin{array}{c} {𝐀}_{fused}=(1-\alpha cdot{𝐀}_{global}+\alpha \cdot {𝐀}_{local} \end{array}$$ (9)

When the image background is extremely complex and the object is small, the network tends to increase α, thereby assigning higher weights to local attention to highlight the object; conversely, it relies more on global attention to suppress macro-level noise.

Next, the fused attention map ${𝐀}_{fused}$ is upsampled to the original spatial resolution (H,W) using bilinear interpolation and multiplied element-wise with the input feature **X** to complete the feature reconstruction and enhancement:


$$\begin{array}{c} 𝐘=𝐗⊗{ℱ}_{up}\left({𝐀}_{fused}\right) \end{array}$$ (10)

Here, ⊗ denotes element-wise multiplication, and ${ℱ}_{up}$ denotes the bilinear upsampling function.

### 2.3 Scale-adaptive WIoU loss function

In the complex background of remote sensing images, target sizes vary greatly, and the images contain a large number of small targets. The IoU values of small targets are highly sensitive to even minor positional shifts in the predicted bounding boxes; even a slight pixel deviation can cause a drastic drop in IoU. This makes small target samples highly prone to generating abnormally high β values during training. In such cases, the traditional WIoUv3 mechanism misclassifies them as “abnormal samples of extremely low quality” and significantly reduces their gradient weights, thereby severely inhibiting the network’s ability to learn features and accurately localize small targets.

To address the issue of imbalanced weight distribution caused by the small-scale IoU mentioned above, this paper proposes the SAWIoU loss function. This loss function introduces a scale-aware factor *S* to dynamically recalibrate the original dissimilarity β. The scale-aware factor *S* is defined as follows:


$$\begin{array}{c} S={\left(\frac{A_{gt}}{\overset{―}{A}+\varepsilon }\right)}^{\eta } \end{array}$$ (11)

Here, $A_{gt}=w_{gt}\cdot h_{gt}$ represents the area of the current ground-truth bounding box; $\overset{―}{A}$ is the exponential moving average of the areas of all ground-truth bounding boxes during training, used to characterize the global average scale of objects in the dataset; η is the scale compensation exponent, used to adjust the compensation for the dispersion of small objects (set to 0.5 in the experiments in this paper); and ε is a minimum constant used to prevent the denominator from becoming zero.

Based on the scale-aware factor, the recalibrated adaptive discretization $\beta_{adj}$ can be expressed as:


$$\begin{array}{c} \beta_{adj}=\beta \cdot S=\frac{L_{IoU}}{\overset{―}{L_{IoU}}}\cdot {\left(\frac{A_{gt}}{\overset{―}{A}+\varepsilon }\right)}^{\eta } \end{array}$$ (12)

From the above equation, it can be seen that when the target is a small object (i.e., $A_{gt}<\overset{―}{A}$), the scale factor is S<1. In this case, the β value—which is abnormally high due to localization sensitivity—is proportionally compressed, allowing it to return to a normal range within the loss function. Conversely, for large objects ($A_{gt}>\overset{―}{A}$) and S>1, their variance is moderately amplified to prevent the model from overfitting to simple, large objects. This adaptive mechanism effectively alleviates the IoU sensitivity disparity caused by differences in target size.

Next, substitute the recalibrated $\beta_{adj}$ into the non-monotonic focusing function to compute the final dynamic gradient gain $r_{SA}$:


$$\begin{array}{c} r_{SA}=\frac{\beta_{adj}}{\delta \cdot \alpha^{\left(\beta_{ad\text{j}}-\delta \right)}} \end{array}$$ (13)

where *α* and *δ* are hyperparameters that control the shape of the non-monotonic focusing curve, and $\beta_{adj}$ denotes the scale-recalibrated adaptive discretization defined in Eq. (12). Finally, by incorporating the distance penalty term $R_{WIoU}$ based on the minimum bounding box into WIoU, the SAWIoU loss function proposed in this paper is fully defined as:


$$\begin{array}{c} L_{SAWIoU}=r_{SA}\cdot R_{WIoU}\cdot L_{IoU} \end{array}$$ (14)

$$\begin{array}{c} R_{WIoU}=exp\left(\frac{(x_{p}-x_{gt}{)}^{\text{2}}+(y_{p}-y_{gt}{)}^{\text{2}}}{(W_{g}^{\text{2}}+H_{g}^{\text{2}}{)}^{*}}\right) \end{array}$$ (15)

In the penalty term $R_{WIoU}$, $\left(x_{p},y_{p}\right)$ and $\left(x_{gt},y_{gt}\right)$ present the center coordinates of the predicted bounding box and the ground-truth bounding box, respectively. $W_{g}$ and $H_{g}$ present the width and height of the smallest bounding rectangle enclosing both. The superscript * indicates that this term is separated during the backpropagation gradient calculation to prevent minor fluctuations in the bounding rectangle dimensions from interfering with the regression of the center points, thereby ensuring the stability of training convergence.

## 3 Experiments

### 3.1 Experimental environment

All experiments were conducted on the Windows 11 operating system using a hardware platform consisting of an Intel Core i9-14900HX CPU, an NVIDIA GeForce RTX 5060 GPU, and 16 GB of RAM. The development environment comprised Python 3.10, CUDA 11.8, and PyTorch 2.7. During the training process, the number of epochs was set to 300, the batch size to 16, the learning rate to 0.01, and the weight decay factor to 5e-4. The SGD optimizer was used for parameter optimization. The YOLOv11n model configuration and pretrained weights released by Ultralytics were used as the baseline implementation.

### 3.2 Description of the datasets

DIOR is a large-scale remote sensing object detection dataset released by Northwestern Polytechnical University. It contains 23,463 images and 20 categories of typical objects (such as aircraft, ships, and vehicles), with all images standardized to a size of 800 × 800 pixels, totaling approximately 190,000 annotated instances. This dataset is large in scale, rich in categories, and diverse in scenarios, covering complex backgrounds such as urban, rural, desert, and marine environments, and containing a large number of small-scale objects. It is suitable for evaluating a model’s comprehensive detection capabilities in complex environments.

Released by Wuhan University, RSOD comprises 976 images and 4 target classes (aircraft, sports fields, overpasses, and oil drums), totaling over 4,500 instances. While the dataset contains relatively concentrated categories, it exhibits significant scale variations with a high proportion of small targets, making it well-suited for evaluating a method’s small-target detection capability in specific scenarios. The dataset was divided into training, validation, and testing subsets at a ratio of 80%/10%/10%, respectively.

### 3.3 Evaluation metrics

To systematically and comprehensively evaluate the performance of the proposed method, this paper assesses it using precision, recall, and the mean average precision. Under a given IoU threshold, precision and recall are defined as:


$$\begin{array}{c} P=\frac{TP}{TP+FP} \end{array}$$ (16)

$$\begin{array}{c} R=\frac{TP}{TP+FN} \end{array}$$ (17)

where precision (P) refers to the proportion of true targets among the predicted positive samples, while recall (R) refers to the proportion of true targets that are correctly detected. *TP* stands for true positive, *FP* for false positive, and *FN* for false negative. The mean precision is defined as:


$$\begin{array}{c} mAP=\frac{\text{1}}{C}\sum_{i=1}^{C}AP_{i} \end{array}$$ (18)

$$\begin{array}{c} AP=\int_{\text{0}}^{\text{1}}P\left(r\right)dr \end{array}$$ (19)

where *P*(*r*) represents the precision at a recall of *r*, *AP* denotes the average precision, and *C* denotes the number of classes. mAP stands for the mean average precision, commonly used evaluation metrics include mAP50 and mAP50:95. Specifically, mAP50 refers to the average value at IoU = 0.5, emphasizing the evaluation of the model’s basic detection capability; whereas mAP50:95 represents the average across multiple IoU thresholds (ranging from 0.5 to 0.95 in 0.05 increments), which places higher demands on localization accuracy and provides a more comprehensive reflection of the model’s overall performance.

### 3.4 Experimental results and analysis

To comprehensively evaluate the detection performance of the method proposed in this paper, we compared it with representative mainstream detection models from recent years using two public remote sensing datasets: DIOR and RSOD. The results are shown in Tables 1, 2. It can be seen that our method achieved an mAP of 0.885 on the DIOR dataset, representing an improvement of 1.2 percentage points over YOLOv11 (0.873) and significantly outperforming remote sensing-specific methods such as CoF-Net (0.758). on the RSOD dataset, the mAP reached 0.971, again outperforming YOLOv11 (0.956) and SSAFNet (0.942). The results demonstrate that our method exhibits consistently superior detection performance across datasets of varying scales and background complexities.

**Table 1:** Performance of the method described in this paper compared to other methods on the DIOR dataset.

Classes	YOLOv5	YOLOv8	YOLOv11	YOLOv12	CoF-Net	SFSANet	SRAFNet	Ours
**Airplane**	0.951	0.954	0.957	0.950	0.840	0.878	0.884	0.965
**Airport**	0.924	0.934	0.948	0.956	0.853	0.833	0.765	0.953
**Baseball field**	0.955	0.951	0.955	0.952	0.826	0.878	0.926	0.954
**Basketball court**	0.932	0.926	0.929	0.937	0.900	0.867	0.879	0.928
**Bridge**	0.523	0.520	0.541	0.562	0.471	0.488	0.358	0.596
**Chimney**	0.923	0.934	0.946	0.938	0.807	0.785	0.838	0.950
**Dam**	0.796	0.766	0.844	0.818	0.733	0.682	0.586	0.847
**Expressway-Service-area**	0.959	0.959	0.979	0.957	0.893	0.850	0.868	0.976
**Expressway-toll-station**	0.833	0.856	0.867	0.851	0.740	0.765	0.668	0.914
**Golf filed**	0.890	0.914	0.926	0.911	0.845	0.732	0.764	0.916
**Ground track field**	0.892	0.889	0.912	0.901	0.832	0.786	0.828	0.920
**Harbor**	0.728	0.764	0.752	0.765	0.574	0.629	0.162	0.750
**Overpass**	0.754	0.777	0.784	0.775	0.622	0.636	0.580	0.796
**Ship**	0.945	0.949	0.949	0.943	0.829	0.902	0.594	0.956
**Stadium**	0.956	0.966	0.971	0.974	0.776	0.834	0.809	0.957
**Storage tank**	0.886	0.889	0.889	0.876	0.682	0.794	0.556	0.895
**Tennis court**	0.961	0.965	0.956	0.964	0.899	0.838	0.906	0.970
**Train station**	0.645	0.647	0.724	0.781	0.687	0.615	0.520	0.841
**Vehicle**	0.610	0.624	0.647	0.623	0.493	0.679	0.532	0.673
**Windmill**	0.887	0.898	0.925	0.914	0.852	0.847	0.910	0.939
**Average mAP50**	0.848	0.854	0.873	0.867	0.758	0.766	0.697	0.885

**Table 2:** Performance of the method described in this paper compared to other methods on the RSOD dataset.

Classes	YOLOv5	YOLOv8	YOLOv11	YOLOv12	SSAFNet	ABNet	CF2PN	ours
**aircraft**	0.966	0.965	0.967	0.985	0.958	0.915	0.955	0.993
**oiltank**	0.984	0.990	0.990	0.988	0.983	0.961	0.994	0.992
**overpass**	0.668	0.802	0.806	0.869	0.846	0.896	0.838	0.907
**playground**	0.949	0.975	0.975	0.983	0.925	0.994	0.957	0.991
**Average mAP50**	0.892	0.933	0.935	0.956	0.928	0.942	0.936	0.971

It is worth noting that our method demonstrates particularly strong performance on classes with complex backgrounds and small object sizes. On the DIOR dataset, the Bridge class achieved an mAP50 of 0.596, representing a 3.4 percentage point improvement over YOLOv12 (0.562) and a 23.8 percentage point improvement over SRAFNet (0.358). For the Train Station class, the mAP50 reached 0.841, an improvement of 6.0 percentage points over YOLOv12 (0.781).

Based on the experimental results described above, the method proposed in this paper achieves optimal or leading detection performance across three publicly available remote sensing datasets, with the most significant improvements observed in complex background classes that are difficult for traditional detectors to handle. Compared to the YOLO series baselines, our method significantly improves detection accuracy while maintaining efficient inference; simultaneously, compared to existing algorithms specifically designed for remote sensing, it demonstrates superior performance across multiple datasets, further validating the superiority and robustness of our proposed method for small-object detection tasks in complex remote sensing backgrounds.

To further evaluate the performance of different methods on small-object detection in complex remote sensing scenes, representative visualization results are presented in Fig 4, while complete visualization comparisons on the DIOR and RSOD datasets are provided in Supporting Information (S1 File). As shown in Fig 4, it can be observed that due to scene complexity and significant variations in object scales, there are notable performance differences among the compared methods in small-object detection on the DIOR dataset. The comparison results indicate that some mainstream methods are prone to missed detections and false detections in areas with dense objects or complex backgrounds, particularly showing prominent shortcomings in small-object categories such as vehicles and bridges. In contrast, the method proposed in this study can detect densely distributed small objects more comprehensively, effectively identifying these weak and small targets while maintaining high localization accuracy. In the RSOD dataset, although the number of categories is relatively limited, there is a high proportion of object scales. The visualization results reveal that traditional methods are prone to localization offsets or redundant detections, such as those of overpasses. However, our method can effectively suppress background interference, achieve more accurate localization of detection boxes, and significantly reduce redundant boxes, thereby providing more compact and accurate detections and demonstrating excellent adaptability.

**Fig 4:**
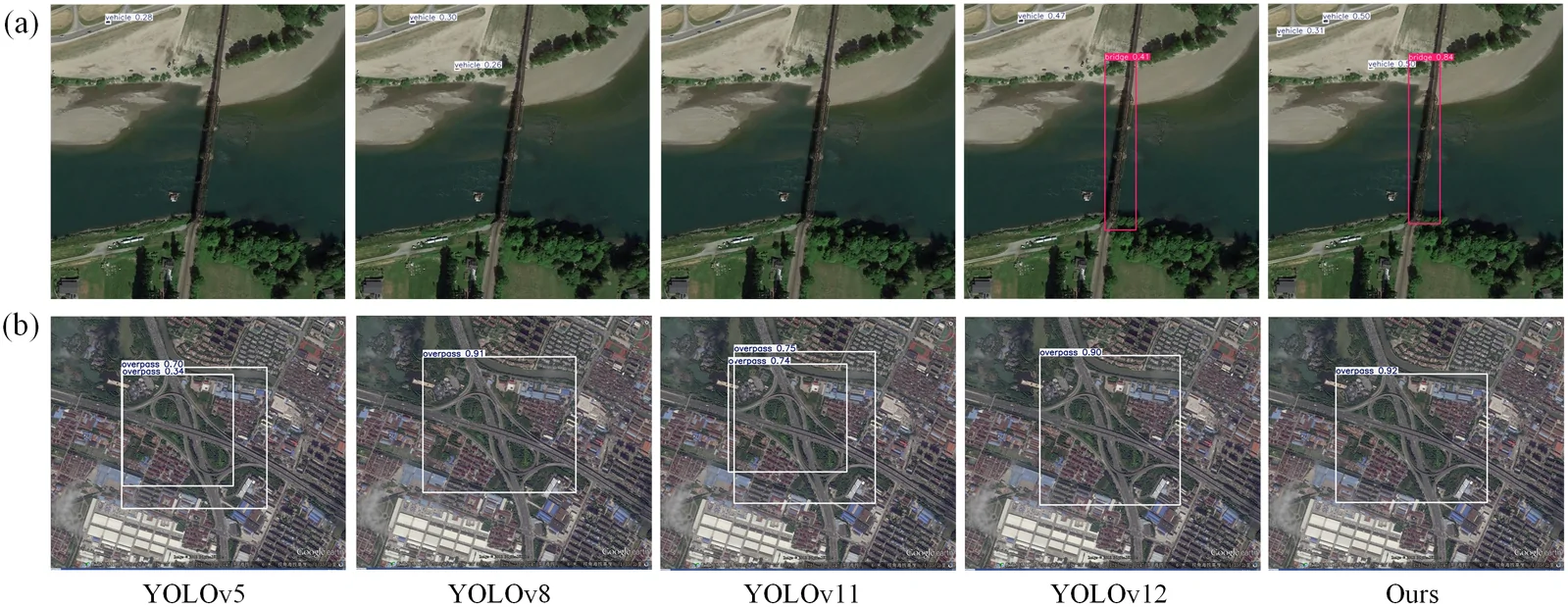
Representative visualization results on different datasets: (a) DIOR datasets, (b) RSOD dataset.

The potential improvements can be attributed to the combined effect of the multi-scale local-global attention mechanism, bidirectional feature fusion strategy, and scale-adaptive loss function, which may jointly enhance feature representation, improve cross-scale interaction, and alleviate regression bias for small objects, thereby contributing to more stable detection behavior in complex scenes.

## 4 Ablation experiments

To verify the effectiveness and necessity of each module in the method proposed in this paper, ablation experiments were conducted on the RSOD dataset, with the results shown in Table 3. The experiments used a baseline model (YOLOv11) without any added modules as a reference, and by sequentially adding each module, we quantitatively evaluated the contribution of each module to detection performance.

**Table 3:** Results of ablation experiments for the proposed method on the RSOD dataset.

MLGA	RCFF	SAWIoU	mAP50	mAP50:95	R	P
			0.946	0.702	0.905	0.956
√			0.953	0.715	0.913	0.958
	√		0.949	0.707	0.907	0.956
		√	0.950	0.709	0.911	0.959
√	√		0.965	0.737	0.929	0.964
	√	√	0.969	0.745	0.930	0.972
√	√	√	0.971	0.768	0.938	0.979

In terms of single-module performance, after introducing MLGA, mAP50 and mAP50:95 improved to 0.953 and 0.715, respectively, while precision increased from 0.956 to 0.958. In contrast, the most significant performance improvement was observed after introducing RCFF, with mAP50 and mAP50:95 reaching 0.949 and 0.707, respectively. Meanwhile, both recall and precision saw notable increases, indicating that the bidirectional feature interaction and recalibration mechanism effectively strengthened multi-scale information fusion, serving as a key factor in enhancing detection performance. After introducing SAWIoU, mAP50 and mAP50:95 increased to 0.95.0 and 0.709, respectively, indicating some improvement in localization accuracy; however, the overall performance improvement was limited, indicating that the effectiveness of loss optimization depends heavily on feature quality.

Further analysis of the dual-module combinations reveals that when MLGA is combined with RCFF, the mAP50:95 further improves to 0.737, with a significant increase in recall, indicating that, based on thorough feature fusion, the attention mechanism can effectively enhance target response. When RCFF is combined with SAWIoU, the mAP50:95 reaches 0.745, but the recall rate decreases slightly, suggesting that loss optimization, while improving localization accuracy, has a certain impact on detection coverage. In contrast, the combination without RCFF shows limited improvement, indicating that thorough multi-scale feature fusion is a prerequisite for performance enhancement.

When all three modules are incorporated simultaneously, the model achieves optimal performance, with mAP50 and mAP50:95 increasing to 0.971 and 0.768, respectively, while the recall rate reaches 0.938 and the precision remains at a high level. The results indicate that MLGA, RCFF, and SAWIoU play distinct roles in feature enhancement, feature fusion, and localization optimization, respectively, and achieve a comprehensive performance improvement through a collaborative mechanism.

## 5 Comparative experiments

To validate the superiority of the feature fusion module proposed in this paper, we compared the performance of different fusion modules on the RSOD dataset using the YOLOv11 framework. The results are shown in Table 4. It can be observed that, compared to the baseline FPN, both BiFPN and Adaptively Spatial Feature Fusion (ASFF) achieve certain improvements in multi-scale information modeling. Specifically, BiFPN enhances the mAP50:95 metric to 0.706 through weighted bidirectional fusion, while ASFF further improves mAP50 to 0.939. However, ASFF shows limited performance gains at high IoU thresholds, indicating insufficient enhancement in fine-grained localization capability. In contrast, the proposed RCFF architecture achieves the best results across all metrics, with mAP50 and mAP50:95 reaching 0.949 and 0.707, respectively. Both recall and precision are simultaneously improved to 0.907and 0.956. These results demonstrate that RCFF effectively strengthens the collaborative representation of shallow-layer details and deep-layer semantics through bidirectional feature interaction and cross-layer recalibration mechanisms, thereby significantly enhancing small object detection and localization performance in complex backgrounds.

**Table 4:** Comparison results of different feature fusion modules on the RSOD dataset.

Feature Fusion Module	mAP50	mAP50:95	R	P
**FPN**	0.946	0.702	0.905	0.956
**BiFPN**	0.947	0.706	0.906	0.950
**ASFF**	0.939	0.701	0.902	0.951
**RCFF**	0.949	0.707	0.907	0.956

Furthermore, to validate the superiority of the feature extraction module proposed in this paper, we built upon the comparative experiments described above and conducted additional comparative experiments by introducing different feature extraction modules within the YOLOv11 + RCFF framework. The results are shown in Table 5. Comparisons reveal that different attention mechanisms all improve model performance to some extent. Among them, SE and CA show comparable performance in terms of mAP50 and mAP50:95, indicating similar effectiveness under the current experimental setting. SimAM achieves slightly higher mAP50:95 compared with SE and CA, demonstrating better performance under high-IoU evaluation. It is worth noting that the MLGA proposed in this paper achieves good results across most metrics, with mAP50 of 0.953, mAP50:95 of 0.715, recall of 0.913, and precision of 0.958. Compared to other attention modules, MLGA achieves improvements of 4.8% (relative to SE and CA) and 2.4% (relative to SimAM) in mAP50:95. These results demonstrate that integrating multi-scale local feature enhancement with global contextual modeling is beneficial to feature discriminability in complex remote sensing scenes.

**Table 5:** Comparison results of the feature extraction module on the RSOD dataset.

Attention Mechanism	mAP50	mAP50:95	R	P
**SE**	0.904	0.667	0.876	0.940
**CA**	0.902	0.659	0.867	0.935
**SimAM**	0.914	0.691	0.89	0.942
**MLGA**	0.953	0.715	0.913	0.958

Building on the comparison of the feature extraction and feature fusion modules described above, we conducted further comparative experiments on different loss optimization strategies within the YOLOv11 + RCFF+MLGA framework. The results are shown in Table 6. It can be observed that distinct performances exist among different IoU loss functions. CIoU achieves a balanced baseline performance, with mAP50 of 0.951 and an mAP50:95 of 0.702. In contrast, GIoU and DIoU exhibit slightly lower mAP50 and recall, indicating a performance gap in bounding box regression relative to CIoU. Although WIoUv3 employs a dynamic focusing mechanism to achieve an mAP50:95 of 0.705, its recall of 0.909 is substantially lower than that of SAWIoU at 0.911. Compared to the aforementioned methods, the proposed SAWIoU achieves optimal results across the majority of metrics, with mAP50 of 0.950, mAP50:95 of 0.709 and recall of 0.911. These results indicate that our method provides a more balanced improvement, achieving superior overall performance on the RSOD dataset.

**Table 6:** Comparison of different loss optimization strategies on the RSOD Dataset.

Loss Optimization Strategy	mAP50	mAP50:95	R	P
**CIoU**	0.946	0.702	0.905	0.956
**GIoU**	0.942	0.70	0.901	0.939
**DIoU**	0.934	0.702	0.894	0.947
**WIOUv3**	0.943	0.705	0.909	0.952
**SAWIoU**	0.950	0.709	0.911	0.959

## 6 Conclusion

To address issues such as insufficient feature representation, inadequate fusion of multiscale information, and limited localization accuracy in the detection of small targets in remote sensing images under complex conditions, this paper proposes a small-target detection method based on feature enhancement and scale-adaptive optimization. First, we achieved full interaction and recalibration of cross-layer features by constructing an RCFF network. Next, we developed the MLGA module to enhance the collaborative modeling of local details and global semantics, and designed SAWIoU to mitigate scale bias in the IoU metric for small objects. This establishes a collaborative improvement mechanism across three levels: feature fusion, feature extraction, and loss optimization. Finally, the system was validated on the DIOR and RSOD datasets. The results demonstrate that the proposed method outperforms or matches the state-of-the-art performance of current mainstream methods across multiple evaluation metrics. Ablation and comparison experiments further validate the independent effectiveness of each module and their synergistic effects, with the RCFF module playing a key role in performance improvement, while MLGA and SAWIoU provide important contributions to feature discrimination and localization accuracy, respectively.

Although the method described in this paper demonstrates good detection accuracy and stability in complex remote sensing scenarios, offering an effective solution for small-object detection, there is still room for further optimization in scenarios with extremely complex backgrounds and ultra-dense small objects. Future research could focus on more refined cross-scale modeling and lightweight design to enhance the method’s generalization ability and practical application value.

## Supporting information

S1 File X. (DOCX)
