# Supplementary material for: A method for detecting small objects in remote sensing images based on feature enhancement and scale-adaptive optimization in complex scenarios
Source: PLoS One. 2026 Oct 27;21(10):e0350873. doi: 10.1371/journal.pone.0350873 (PMC13712719; doi:10.1371/journal.pone.0350873)
Supplement: Supplementary file 1 [file pone.0350873.s001.docx]

Fig S1 and Fig S2 present visualization comparisons of different detectors on the DIOR and RSOD datasets. Due to scene complexity and scale variations, these methods show varying performance in remote sensing small-object detection.

As shown in Fig S1 for the DIOR dataset, which contains complex urban-rural mixed scenes with significant scale variations, the compared methods exhibit clear differences. Mainstream detectors often suffer from missed detections or incomplete localization for small or occluded objects such as bridges and vehicles in cluttered backgrounds. Our method, however, consistently identifies most small and densely distributed objects across diverse scenes. In the RSOD dataset shown in Fig S2, some compared methods exhibit localization deviations or redundant detections, such as those of overpasses or structured regions. Our method, by contrast, maintains stable performance across scales, effectively suppressing background interference for both small and large objects.

Overall, these results demonstrate that our method achieves robust and consistent detection across diverse remote sensing scenarios, showing strong adaptability to complex backgrounds and scale variations.

Fig S1. Visualization results of different methods on the DIOR dataset.

Fig S2. Visualization results of different methods on the RSOD dataset.
